# Supplementary figures and images for: In situ study of environmental factors (temperature and salinity) affecting cohort patterns and growth rates in Ciona robusta
Source: PeerJ. 2025 Sep 18;13:e20034. doi: 10.7717/peerj.20034 (PMC12450370; doi:10.7717/peerj.20034)

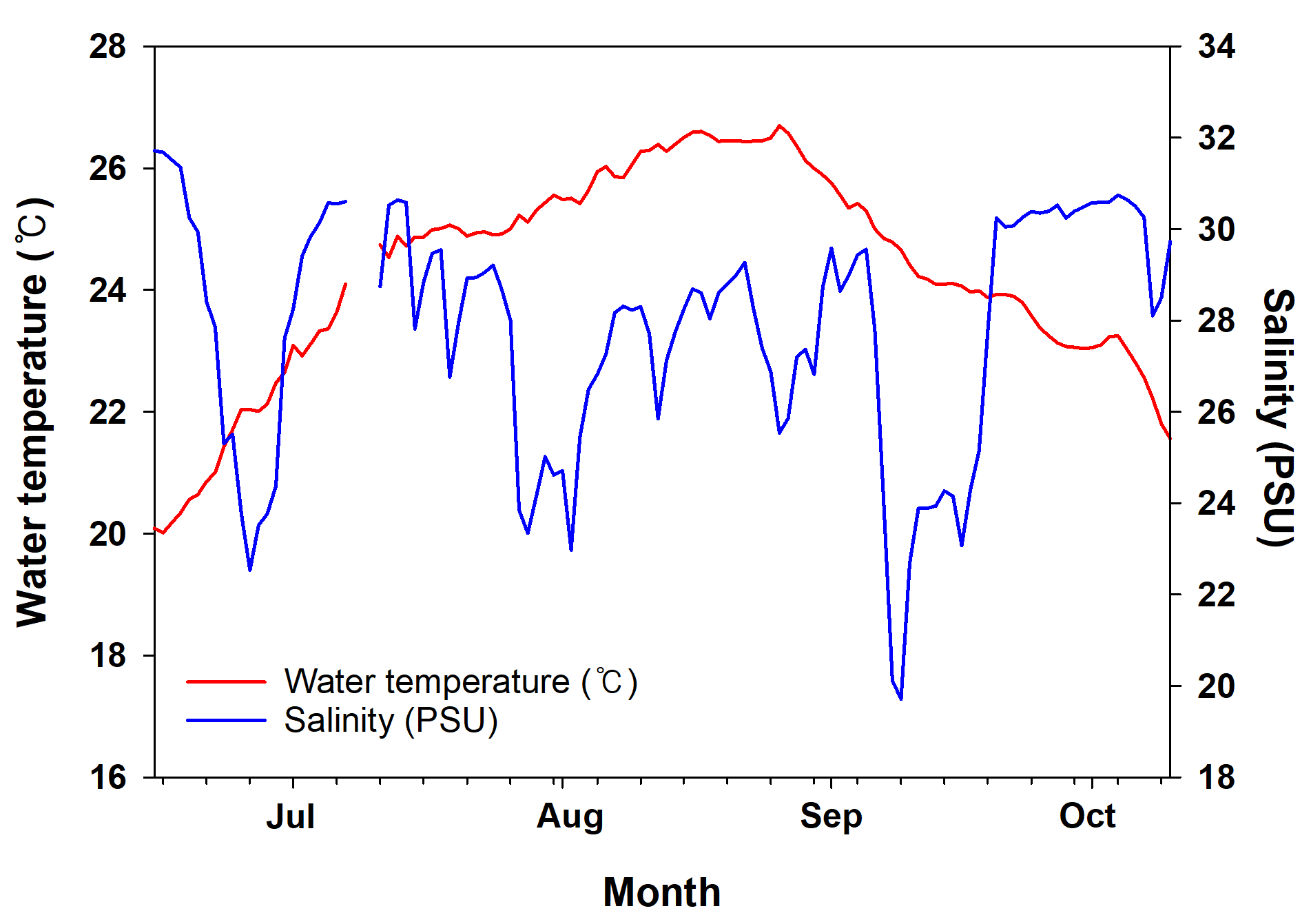

Supplement: Supplemental Information 1 — Mean daily water temperature (red line) and salinity (blue line) in Mokpo during the survey period. [file peerj-13-20034-s001.png]

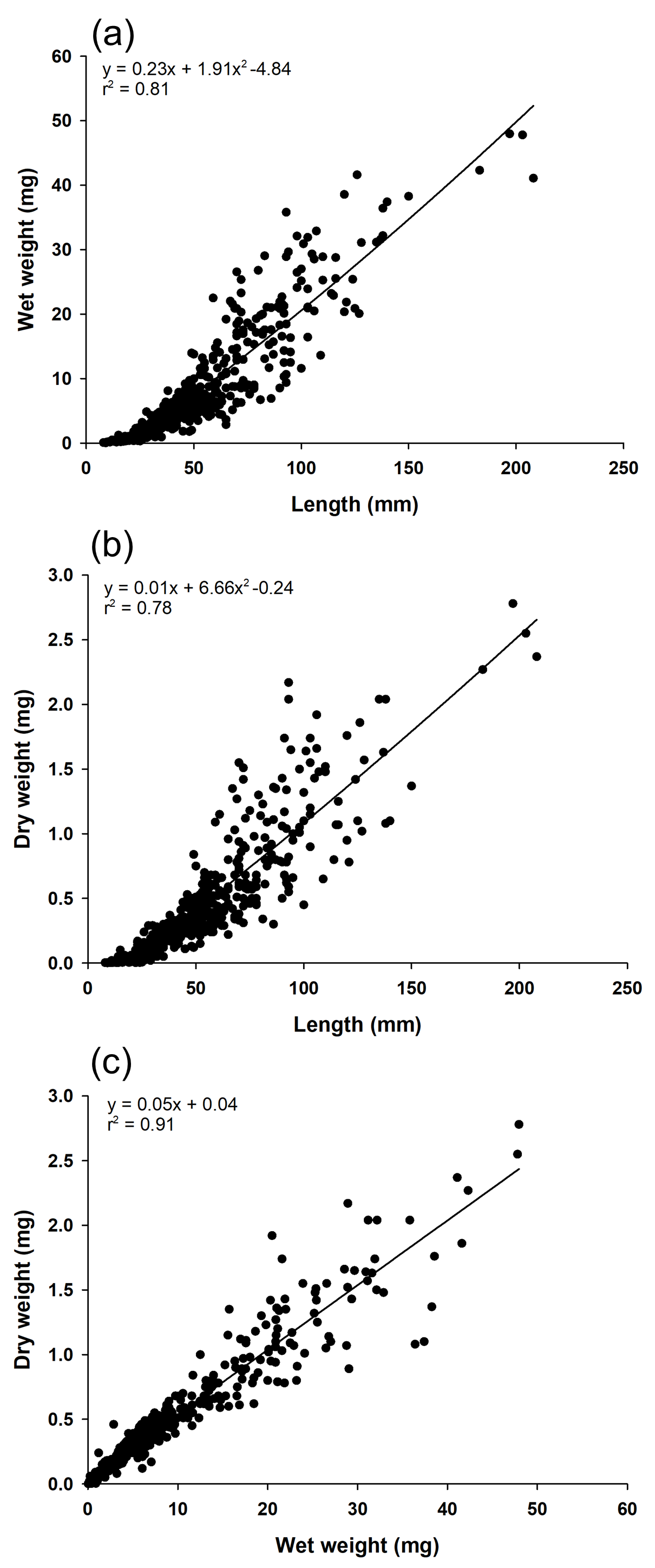

Supplement: Supplemental Information 2 — Correlations between morphological measurements. correlation between body length and wet weight (A), correlation between body length and dry weight (B), and correlation between wet weight and dry weight (C). Each plot shows the regression equation and coefficient of determination (r2). [file peerj-13-20034-s002.png]
