# Supplementary material for: In situ study of environmental factors (temperature and salinity) affecting cohort patterns and growth rates in Ciona robusta
Source: PeerJ. 2025 Sep 18;13:e20034. doi: 10.7717/peerj.20034 (PMC12450370; doi:10.7717/peerj.20034)
Supplement: Supplemental Information 3 — Pairwise t-test results comparing different cohorts (C1 –C5) across multiple surveys, showing test statistics, degrees of freedom (df), p-values, and 95% confidence intervals. Bold text indicates statistical significance (p < 0.05). [file peerj-13-20034-s003.docx]

| Survey number | Cohort | | Statistic | df | *p* | 95% Confidence interval | |
| --- | --- | --- | --- | --- | --- | --- | --- |
|  |  |  |  |  |  | Lower | Upper |
| 1 | C1 | C3 | 22.3019 | 116 | **0.0000** | 27.6182 | 33.0018 |
|  | C1 | C2 | 29.0398 | 116 | **0.0000** | 54.3051 | 62.2549 |
|  | C2 | C3 | 16.5012 | 116 | **0.0000** | 24.6128 | 31.3272 |
| 2 | C1 | C2 | 16.1523 | 102 | **0.0000** | 20.0616 | 25.6784 |
|  | C1 | C3 | 29.1463 | 102 | **0.0000** | 46.9142 | 53.7658 |
|  | C1 | C4 | 45.0524 | 102 | **0.0000** | 75.6366 | 82.6034 |
|  | C2 | C3 | 18.0638 | 102 | **0.0000** | 24.4537 | 30.4863 |
|  | C2 | C4 | 36.2059 | 102 | **0.0000** | 53.1684 | 59.3316 |
|  | C3 | C4 | 15.6266 | 102 | **0.0000** | 25.1269 | 32.4331 |
| 3 | C2 | C3 | 19.1446 | 142 | **0.0000** | 29.6015 | 36.4185 |
|  | C2 | C4 | 32.7304 | 142 | **0.0000** | 66.9467 | 75.5533 |
|  | C3 | C4 | 21.7062 | 142 | **0.0000** | 34.7574 | 41.7226 |
| 4 | C2 | C3 | 16.6036 | 104 | **0.0000** | 39.9425 | 50.7775 |
|  | C2 | C4 | 32.3256 | 104 | **0.0000** | 88.149 | 99.671 |
|  | C3 | C4 | 17.8321 | 104 | **0.0000** | 43.1509 | 53.9491 |
| 5 | C3 | C4 | 33.7422 | 116 | **0.0000** | 52.6187 | 59.1813 |
|  | C3 | C5 | 70.3871 | 116 | **0.0000** | 92.4726 | 97.8274 |
|  | C4 | C5 | 26.6176 | 116 | **0.0000** | 36.3294 | 42.1706 |
| 6 | C4 | C5 | 19.9554 | 118 | **0.0000** | 46.6867 | 56.9733 |
