# Supplementary material for: In situ study of environmental factors (temperature and salinity) affecting cohort patterns and growth rates in Ciona robusta
Source: PeerJ. 2025 Sep 18;13:e20034. doi: 10.7717/peerj.20034 (PMC12450370; doi:10.7717/peerj.20034)
Supplement: Supplemental Information 4 — Growth rates (r) of various cohorts (C1 –C5) measured as of the survey date from June to October 2022. [file peerj-13-20034-s004.docx]

| Cohort | Date | Survey number | Growth rate (r) |
| --- | --- | --- | --- |
| C1 | Jun. 26. 2022 | 1 |  |
|  | Jul. 9. 2022 | 2 | -0.0353 |
| C2 | Jun. 26. 2022 | 1 |  |
|  | Jul. 9. 2022 | 2 | 0.0073 |
|  | Jul. 23. 2022 | 3 | 0.3384 |
|  | Aug. 5. 2022 | 4 | 0.2829 |
| C3 | Jun. 26. 2022 | 1 |  |
|  | Jul. 9. 2022 | 2 | 0.0230 |
|  | Jul. 23. 2022 | 3 | 0.4218 |
|  | Aug. 5. 2022 | 4 | 0.2659 |
|  | Aug. 20. 2022 | 5 | 0.3156 |
| C4 | Jul. 9. 2022 | 2 |  |
|  | Jul. 23. 2022 | 3 | 0.6241 |
|  | Aug. 5. 2022 | 4 | 0.2986 |
|  | Aug. 20. 2022 | 5 | 0.4874 |
|  | Sep. 3. 2022 | 6 | 0.2774 |
| C5 | Aug. 20. 2022 | 5 |  |
|  | Sep. 3. 2022 | 6 | 0.2767 |
|  | Sep. 17. 2022 | 7 | 0.2973 |
|  | Oct. 1. 2022 | 8 | 0.0136 |
