# Supplementary material for: In situ study of environmental factors (temperature and salinity) affecting cohort patterns and growth rates in Ciona robusta
Source: PeerJ. 2025 Sep 18;13:e20034. doi: 10.7717/peerj.20034 (PMC12450370; doi:10.7717/peerj.20034)
Supplement: Supplemental Information 5 — Multiple linear regression results for the effect of water temperature and salinity on growth rate for each cohort . Model summary: adjusted r 2 = 0.438, F (2, 17) = 8.413, p = 0.002 . Bold indicates significant values (p < 0.05). [file peerj-13-20034-s005.docx]

| Variable | Estimate | Std. Error | t | *p* |
| --- | --- | --- | --- | --- |
| (Intercept) | -1.043 | 0.423 | -2.462 | **0.024** |
| Water temperature | 0.079 | 0.019 | 4.050 | **0.000** |
| Salinity | -0.024 | 0.014 | -1.665 | 0.114 |
