# Supplementary material for: In situ study of environmental factors (temperature and salinity) affecting cohort patterns and growth rates in Ciona robusta
Source: PeerJ. 2025 Sep 18;13:e20034. doi: 10.7717/peerj.20034 (PMC12450370; doi:10.7717/peerj.20034)
Supplement: Supplemental Information 6 — Results of normality (Shapiro–Wilk test) and homogeneity of variance (Levene Test) tests for water temperature and salinity data. [file peerj-13-20034-s006.docx]

| Groups | Shapiro-Wilk test | |  | Levene Test | | | |
| --- | --- | --- | --- | --- | --- | --- | --- |
|  | W^1^ | *p* |  | df1 | df2 | F^2^ | *p* |
| Water temperature | 0.9626 | 0.6540 |  | 7 | 12 | 1.6699 | 0.2076 |
| Salinity | 0.9314 | 0.2062 |  |  |  |  |  |
